# Sleeve Gastrectomy Versus Banded Roux-en-Y Gastric Bypass for Obesity and Diabetes Mellitus: Psychology and Quality of Life Outcomes at 10 Years

**DOI:** 10.1007/s11695-026-08623-3

**Published:** 2026-04-08

**Authors:** Preekesh Suresh Patel, Megan Grinlinton, Anamitra Nair, Jack Pullman, Lindsay D Plank, Rinki Murphy, Michael Booth

**Affiliations:** 1 Department of General Surgery - Upper Gastrointestinal/Bariatric Unit, Te Whatu Ora Health New Zealand - Waitemata, Auckland, New Zealand; 2 Faculty of Medical and Health Sciences, University of Auckland, Auckland, New Zealand; 3 Department of Endocrinology, Te Whatu Ora Health New Zealand - Counties Manukau, Auckland, New Zealand

## Abstract

**Background:**

Obesity impacts physical, physiological and psychological domains of life. The long-term effects of metabolic bariatric surgery (MBS) on patient-centred outcomes such as mental health and quality of life (QOL) may enhance the management of obesity and the application of MBS.

**Methods:**

A prospective, blinded, double arm, parallel, randomised trial was carried out at a single bariatric centre in Waitemata, New Zealand. Adults with type 2 diabetes mellitus and obesity were randomised 1:1 to undergo laparoscopic sleeve gastrectomy (SG) or silastic ring Roux-en-Y gastric bypass (SR-RYGB). After unblinding at 5 years, patients were followed up at 10 years. This analysis focuses on secondary outcomes: Hospital Anxiety and Depression Scale (HADS) scores and RAND-36 QOL scores.

**Results:**

Of 114 patients randomised, 80 patients (70.2%) completed 10-year follow up (39 SG; 41 SR-RYGB). SR-RYGB was associated with greater weight loss (33.3 kg vs. 25.8 kg, *p* = 0.031) and trended towards higher diabetes remission (31.7% vs. 23.1%, adjusted OR 2.07, 95% CI 0.70, 6,10, *p* = 0.186). Mean HADS anxiety score for SG decreased by 1.59 units over 10-years (*p* = 0.027). Improvements in RAND-36 scores were significantly greater after 10 years for SR-RYGB than SG for physical function (19 vs. 12 points, *p* = 0.026) and general health (17 vs. 10 points, *p* = 0.036). Role limitation due to emotional problems worsened significantly by 16 points for the SG group (*p* = 0.032).

**Conclusion:**

SR-RYGB demonstrated superior long-term improvements in physical function and general health QOL domains of RAND-36 compared to SG. Anxiety symptoms may be reduced long-term following SG.

## Key Points

- SG led to improved long-term anxiety symptom scores.
- SG and SR-RYGB both had improved physical function and general health QOL scores.
- SR-RYGB is superior to SG for improvement in long-term QOL scores.

## Introduction

As our understanding of obesity grows, it becomes clearer that the interplay between obesity, its treatments and its impact on every organ system is complex. Psychological health and quality of life (QOL) are patient-centred factors that are impacted by obesity and metabolic bariatric surgery (MBS) [1–3]. MBS has the potential to improve psychological health and QOL with weight loss and resolution of obesity associated medical problems [2, 4–6]. On the contrary, the adverse outcomes of MBS such as complications, altered gastrointestinal function and psychosocial implications can negatively impact psychology and QOL [7–9]. The type of MBS performed may also impact psychology and QOL differentially [10].

Funding for MBS varies globally and, in New Zealand (NZ), despite consensus guidelines recommending improved public funding/access, it is predominantly privately funded [11, 12].

Given MBS in the public sector is a finite resource, it should be reserved for those that will derive the most benefit. Psychological health and QOL are parameters that are currently underutilised in determining access to public MBS.

Psychological health has strong emphasis in the preoperative multidisciplinary work-up for MBS. Preoperative psychological screening is recommended by the International Federation for Surgery of Obesity (IFSO) to identify optimisable psychosocial factors to improve patient’s ability to both cope with and achieve maximal benefit from MBS [13]. Furthermore, symptoms of some psychological disorders can overlap with those experienced after MBS (e.g. changes in appetite and bowel habit). The long-term interplay between psychological symptoms and MBS outcomes are poorly understood.

There has been a stronger focus on patient-centred QOL outcomes following MBS to provide insights into surgical efficacy that are reflective of patient’s goals of obesity treatment [5, 14]. Questionnaire based surveys (e.g. RAND-36) allow this to be performed and reported in a standardised and comparable fashion [15]. QOL information could guide a more individualised approach to MBS selection.

QOL and psychology outcomes have previously been reported for this randomised trial comparing sleeve gastrectomy (SG) and silastic-ring Roux-en-Y gastric bypass (SR-RYGB) in patients with obesity and type 2 diabetes mellitus (T2DM) in NZ [16, 17]. Depressive and anxiety symptoms improved significantly within the first year after both types of surgery. At five years, only depressive symptoms were lower than baseline, while anxiety symptoms returned to baseline levels by year five. This study at 10 years adds to the existing high quality randomised studies that report long term outcomes comparing SG to Roux-en-Y gastric bypass (RYGB) given it considers silastic ring augmented RYGB (demonstrating greater weight loss and limited weight recidivism), and focuses on patient-centred outcomes [18–20].

The objectives of this paper were to compare SG and SR-RYGB in patients with obesity and T2DM; to identify long-term (10-year postoperative) changes in psychological health and quality of life; and to evaluate whether preoperative depression and anxiety symptoms influence long-term weight loss.

## Methods

### Ethics

Local/regional ethics approval was obtained in 2011. The trial was registered with the appropriate national registry. Follow-up extension beyond five years was granted.

### Design

This was a prospective, double-armed, parallel, randomised trial that was double-blinded (patient and assessor) to five-years. The full study protocol was published previously [21]. The setting was a single bariatric centre in Waitemata, NZ. Patients referred for consideration for MBS were screened based on their referral (from general practitioners and hospital specialists). Eligible patients attended an educational seminar including presentations on obesity and MBS delivered by our multidisciplinary team (surgical fellow, dietitian and clinical nurse specialist). Selection criteria application and measurement of baseline anthropometrics (height and weight) were performed at their first specialist appointment.

### Selection Criteria

Inclusion criteria included: age 20–55, body mass index (BMI) 35–65 kg/m^2^ (> 5-year duration), diagnosis of T2DM (made at least six-months prior), suitability to undergo SG or SR-RYGB, ability to provide informed consent and commitment to five-years of follow-up. Exclusion criteria included: type 1 diabetes, secondary diabetes, oral steroid use, cigarette smoking, history of chronic pancreatitis, pregnancy, post-prandial C-peptide < 350pmol/L and unable to safely receive general anaesthesia.

### Randomisation

Computer generated randomisation was performed with allocation concealment and minimisation. Minimisation was based on age (20–30, 30–40, 40–50 years), ethnicity (Māori, Pacific, European or other), BMI (35-44.9, 45-54.9, 55–65 kg/m^2^), time since T2DM diagnosis (< 5 years, 5–10 years, > 10 years) and insulin requirement (yes, no).

### Interventions

Details of SG and SR-RYGB techniques used have been published [22]. Procedures were performed laparoscopically. Orogastric bougies were used to calibrate both SG (36 F) and the SR-RYGB gastric pouch (32 F). SR-RYGB limb lengths were 50 cm (biliopancreatic) and 100 cm (antecolic roux limb). The silastic ring for SR-RYGB was 6.5 cm and secured 2 cm proximal to the gastrojejunostomy.

### Outcomes

This analysis focuses on secondary outcomes; psychological symptoms and QOL at 10 years postoperatively. The psychological symptoms were assessed using the hospital anxiety and depression scale (HADS) [23]. This is a validated screening tool based on 14 questions (7 for depression and 7 for anxiety), each scored from 0 to 3. A HADS depression score of ≥8 indicated mild to severe depressive symptoms. A HADS anxiety score of ≥8 indicated mild to severe anxiety symptoms. Scores of ≤7 indicated an absence of symptoms. QOL was evaluated using the validated RAND-36 score, based on 8 overarching domains: physical functioning (PF), role limitation (physical), bodily pain, general health (GH), role limitation (emotional) (RE), energy/fatigue, emotional well-being and social functioning [15]. Each domain is scored from 0 indicating poor health to 100 indicating good health.

### Sample Size and Statistical Analysis

The original sample size was calculated to detect a 29% difference in T2DM remission between the SR-RYGB group (assumed 88% remission) and SG group (assumed 59% remission) using a 2-sided statistical test with 80% power and an alpha risk of 0.05. While this paper focuses on secondary outcomes, the same cohort was used for longitudinal analysis of psychological and QOL measures. Normally distributed continuous variables are reported as means and standard deviations (SD) and Student’s t-test was used for between-group comparisons. Categorical data were compared between groups using Fisher’s exact test. The difference in proportion of participants achieving diabetes remission was compared between SR-LRYGB and LSG using logistic regression with adjustment for stratification variables. Repeated measures mixed effects models with adjustment for stratification variables were used to analyse HADS, RAND-36 and weight changes from baseline. Least squares means with standard errors (SEs) were plotted graphically. A two-sided P value < 0.05 was considered to indicate statistical significance. Analyses were performed using SAS version 9.4 statistical software (SAS Institute, Cary, NC).

## Results

**Fig. 1:**
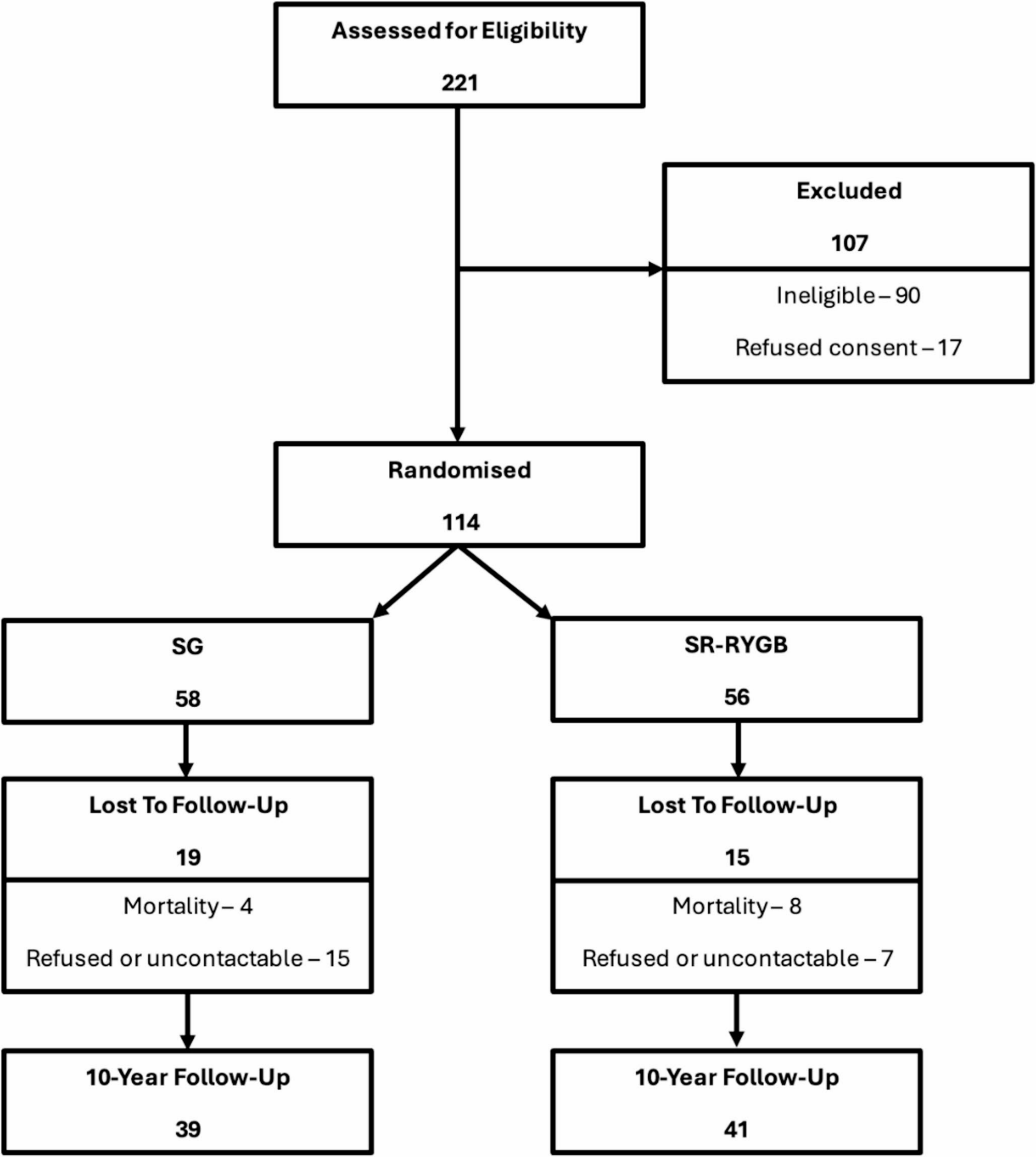
Summary of participant flow and follow-up for psychological symptom and quality of life outcomes

A total of 114 patients were recruited and their progression through the study is summarised in Fig. 1. At 10 years, 80 (41 SR-RYGB, 39 SG) patients had completed HADS and QOL questionnaires, giving a follow-up completion rate of 70.2%. Of those that did not complete 10-year follow-up, there were 22 patients that either declined further trial participation or were uncontactable and 12 deaths (four SR-RYGB, eight SG). Of the mortalities (all of which were not directly relatable to bariatric surgery): six were related to advanced malignancy (metastatic renal cell carcinoma (x2), glioblastoma multiforme, metastatic cholangiocarcinoma, malignant small bowel obstruction and metastatic endometrioid adenocarcinoma), two were cardiac in nature (cardiac arrest and heart failure) and one was a trauma (motor vehicle accident). The cause of death was unknown for three participants. The baseline characteristics are summarised in Table 1, demonstrating no significant differences between those that did and did not complete 10 years of follow-up. Mild to moderate depressive symptoms (scores of ≥8) at 10 years were present for 4 (10.3%) SG patients and 6 (14.6%) SR-RYGB patients. Mild to moderate anxiety symptoms (scores of ≥8) at 10 years were present for 8 (20.5%) SG patients and 9 (22.0%) SR-RYGB patients. Baseline emotional wellbeing scores were significantly greater in the SR-RYGB by chance. All other baseline characteristics were balanced amongst both groups, with the whole cohort on average being predominantly NZ European, aged in their forties with a BMI in the forties and a similar number of patients with pre-existing psychological symptoms.

**Table 1:** Baseline characteristics for those that completed and those not completing HADS and QOL data questionnaires at 10 years follow-up. ^*^Comparison of SG and SR-RYGB in completed group; ^‡^Comparison of groups completing and not completing follow-up

	Completed				Not completed	
	Total (*n* = 80)	SG (*n* = 39)	SR-RYGB (*n* = 41)	*p*-value*	Total (*n* = 34)	*p*-value^‡^
Age (years), mean (SD)	47.5 (6.1)	46.9 (6.5)	47.9 (5.7)	0.466	45.4 (7.5)	0.130
Female, *n* (%)	38 (47.5)	15 (38.5)	23 (56.1)	0.125	21 (61.8)	0.219
Ethnicity, *n* (%)				0.246		0.590
New Zealand European	51 (63.8)	28 (71.7)	23 (56.1)		21 (61.8)	
Māori	12 (15.0)	5 (12.8)	7 (17.1)		8 (23.5)	
Pacific	7 (8.8)	1 (2.6)	6 (14.6)		3 (8.8)	
Other	10 (12.5)	5 (12.8)	5 (12.2)		2 (5.9)	
Duration of type 2 diabetes (y), *mean (SD)*	7.2 (5.5)	6.7 (5.1)	7.7 (5.8)	0.410	6.1 (4.7)	0.300
HbA1c (mmol/mol), *mean (SD)*	62.9 (14.6)	62.2 (11.1)	63.6 (17.4)	0.673	64.1 (17.7)	0.718
Weight (kg), *mean (SD)*	125.6 (22.9)	125.5 (22.9)	125.7 (23.2)	0.965	128.3 (26.2)	0.608
BMI (kg/m^2^), *mean (SD)*	42.5 (6.6)	42.1 (6.3)	42.8 (6.8)	0.641	44.1 (6.4)	0.224
HADS scores						
Depression, *mean (SD)*	3.5 (3.0)	3.6 (3.2)	3.4 (2.9)	0.790	4.0 (3.9)	0.478
Anxiety, *mean (SD)*	5.5 (4.1)	6.1 (4.6)	4.8 (3.5)	0.155	5.9 (4.4)	0.633
HADS Depression Score ≥ 8, *n* (%)	11 (13.8)	6 (15.4)	5 (12.2)	0.753	7 (20.6)	0.404
HADS Anxiety Score ≥ 8, *n* (%)	22 (27.5)	12 (30.8)	10 (24.4)	0.619	11 (32.4)	0.654
RAND-36 scores, *mean (SD)*						
Physical functioning	70.0 (24.4)	73.8 (22.4)	66.4 (25.9)	0.179	66.9 (23.3)	0.534
Role limitations due to physical health	72.2 (36.0)	73.7 (35.8)	70.7 (36.6)	0.718	63.6 (44.7)	0.291
Role limitations due to emotional problems	86.1 (24.0)	84.2 (29.8)	87.8 (26.6)	0.572	78.8 (38.0)	0.264
Energy/fatigue	59.3 (19.6)	59.0 (19.1)	59.6 (20.4)	0.896	54.2 (21.3)	0.233
Emotional Well Being	80.9 (16.4)	77.0 (18.8)	84.6 (12.9)	0.041	78.4 (16.6)	0.465
Social Functioning	82.3 (21.8)	81.4 (21.0)	83.2 (22.6)	0.711	80.3 (25.0)	0.665
Bodily Pain	74.1 (22.7)	75.0 (22.4)	73.2 (23.2)	0.725	71.4 (24.9)	0.586
General Health	54.1 (24.0)	57.5 (23.3)	50.9 (24.6)	0.219	48.9 (25.3)	0.310

Total weight loss over 10 years was superior for SR-RYGB over SG at 33.3 (SE 2.4) kg vs. 25.8 (2.5) kg (*p* = 0.031). Diabetes remission at 10 years was seen in 31.7% (13 of 41 participants) in the SR-RYGB group compared with 23.1% (9 of 39 participants) in the SG group (adjusted OR 2.07; 95% CI 0.70, 6, 10; *P* = 0.186).

### HADS

**Fig. 2:**
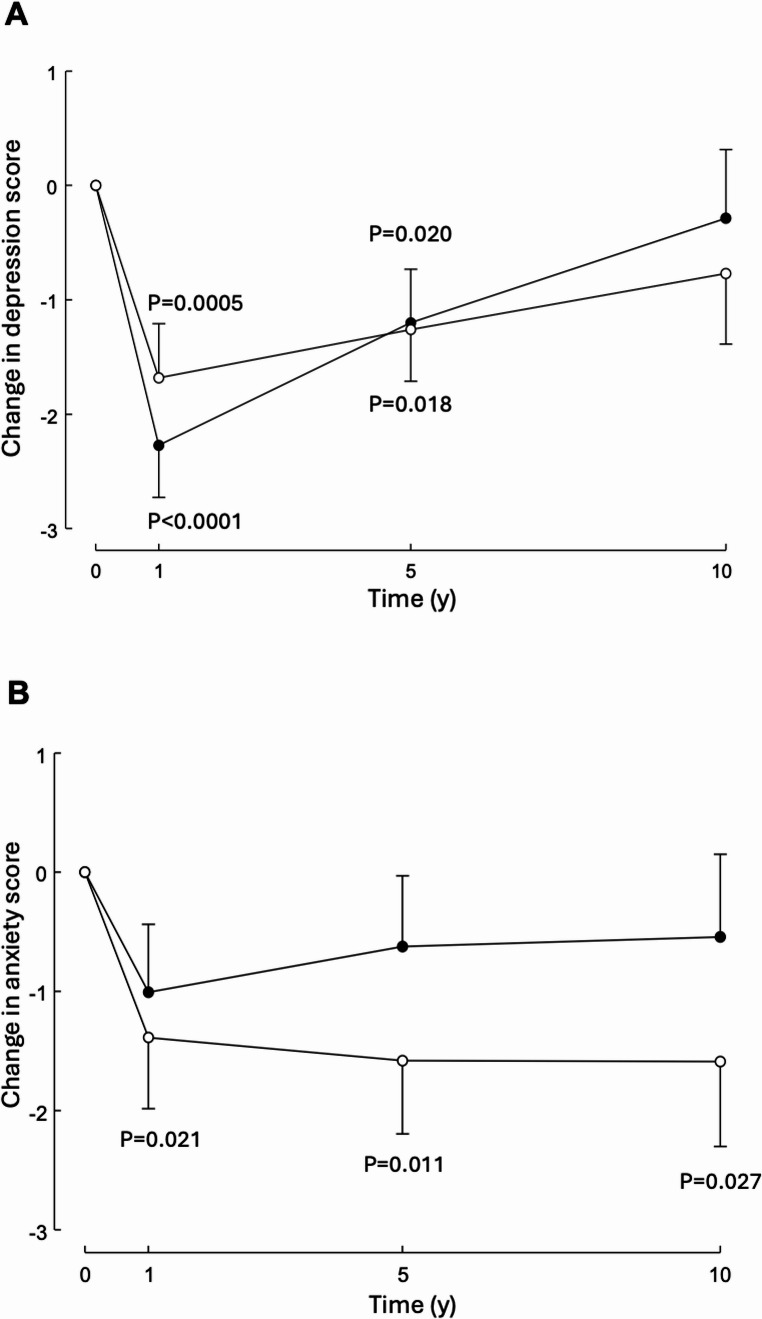
Change in depression (**A**) and anxiety (**B**) scores over 10 years in patients undergoing SG (open symbols) or SR-RYGB (closed symbols). Data are mean ± SE. P values relate to changes from baseline

HADS depression symptom scores improved for both groups at 1 year (-1.68 (SE 0.47), *P* = 0.0005, for SG; -2.27 (0.45), *P* < 0.0001, for SR-RYGB) and 5 years follow-up (-1.26 (SE 0.53), *P* = 0.018, for SG; -1.20 (0.51), *P* = 0.020, for SR-RYGB) but there was no significant change from baseline by 10 years follow-up for either group (-0.77 (0.62), *P* = 0.215, for SG; -0.29 (0.60), *P* = 0.634, for SR-RYGB) (Fig. 2). Mean score for anxiety decreased at 1-year (-1.39 (0.60), *P* = 0.021) and 5-year follow-up in SG patients (-1.58 (SE 0.61), *P* = 0.011) and remained below baseline at 10 years (-1.59 (0.71), *P* = 0.027). For SR-RYGB patients, mean anxiety scores did not change from baseline at 1 year (-1.01 (0.57), *P* = 0.079), 5 years (-0.62 (0.59), *P* = 0.295) and 10 years (-0.54 (0.69), *P* = 0.435) (Fig. 2). There were no significant between-group differences.

**Fig. 3:**
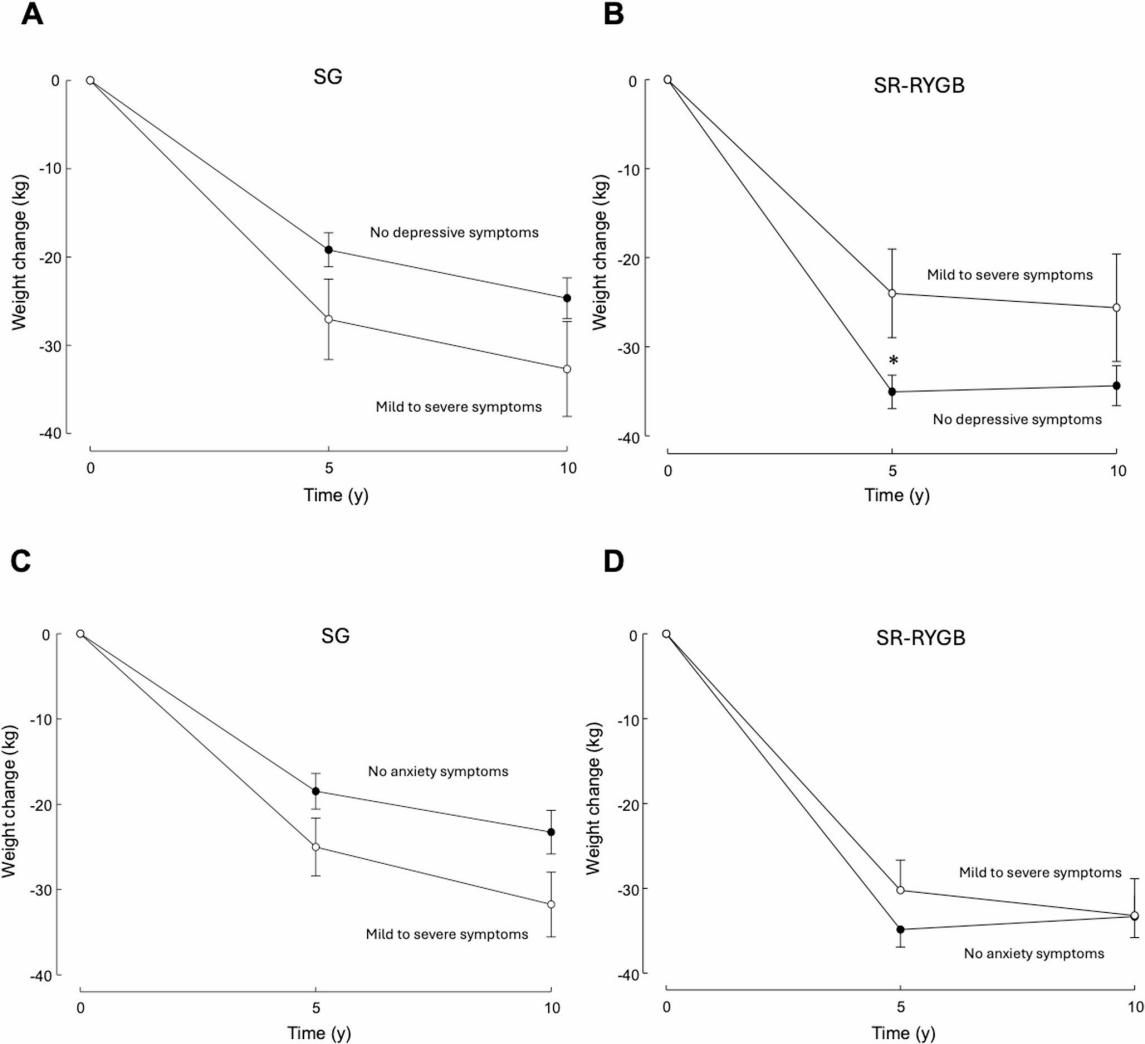
Total weight change over 10 years based on operative type and the presence/absence of baseline depressive (**A **and **B**) and anxiety (**C **and **D**) symptoms. No symptoms (closed symbols); mild or severe symptoms (open symbols). Data are mean ± SE. * *P* = 0.044

The relationships between psychological outcomes, surgery type and weight loss at 5 and 10 years are shown in Fig. 3. Patients who underwent SG with baseline depressive or anxiety symptoms had similar total weight loss at 10 years to those without symptoms (32.7 (9.5) kg vs. 24.5 (2.4) kg, *p* = 0.437 and 31.8 (4.7) kg vs. 23.0 (2.8) kg, *P* = 0.128, respectively). Patients who underwent SR-RYGB with baseline depressive or anxiety symptoms had similar weight loss at 10 years to those without symptoms (25.6 (7.1) kg vs. 34.4 (2.5) kg, *P* = 0.229 and 33.2 (4.8) kg vs. 33.3 (2.8) kg, *P* = 0.981, respectively).

### RAND-36

**Fig. 4:**
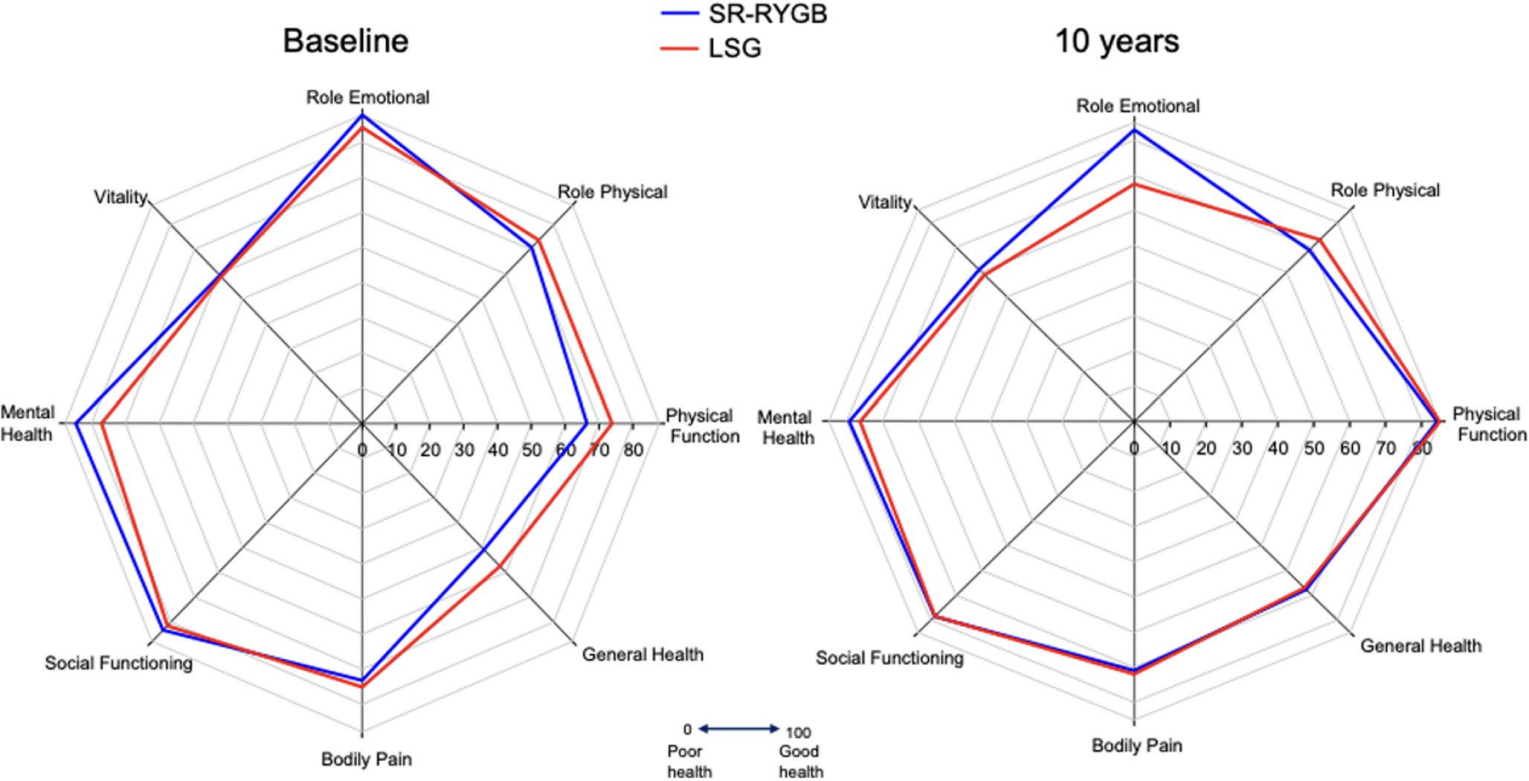
SG and SR-RYGB RAND-36 quality of life scores at baseline and 10 years

RAND-36 scores are depicted in Fig. 4. PF scores improved significantly for each group over time (SG: 12 points, *P* = 0.004 and SR-RYGB 19 points, *P* < 0.0001) with a significant between group difference (*P* = 0.026). GH scores also improved significantly for each group over time (SG: 10 points, *P* = 0.018 and SR-RYGB: 17 points, *P* < 0.0001) with a significant difference between the groups (*P* = 0.036). Although RE scores worsened significantly for the SG group (16 points, *P* = 0.032) long-term, those for SR-RYGB did not and there was no significant between-group difference. For all other RAND-36 domains there were no significant changes over time and no significant between-group differences.

## Discussion

The findings address the objectives of providing a comparison of SG and SR-RYGB focusing on psychological symptoms and QOL at 10 years postoperatively. They provide an extension to the published five-year results [17].

Depression symptom scores for SG and SR-RYGB were similar to baseline levels at 10 years, with a significant improvement noted at the one and five-year mark. Anxiety symptom scores significantly improved for SG at 10 years. Anxiety symptom scores remained similar for SR-RYGB across the follow-up period. There were no significant between-group differences.

The short-term psychological symptom score improvement demonstrated by our cohort supports the existing literature with similar findings [2, 17, 24, 25]. This likely is contributed to by weight loss, resolution of associated medical problems and improved body image.

The long-term results demonstrate improved depression and anxiety symptom scores from baseline for both groups. Aside from SG anxiety scores having sustained improvement from years five to 10, most 10-year results either stagnate or worsen compared to five-year results. This may be contributed to by recurrent weight gain, relapse of associated medical issues, nutrient deficiencies and requirement for either long-term medications and/or further procedures. The long-term data that exists regarding psychological symptoms following MBS are heterogeneous (method of measurement, procedures included, definitions of “long-term”) and because psychological/QOL parameters are generally not the primary outcome, this has implications on study power and result generalisability [26–29]. The general trend is an improvement in depression and anxiety scores, which is echoed within our findings.

The greatest overall improvement in psychological symptom scores was the 1.59-point reduction in anxiety scores for SG patients which may be related to the generally lower “side effect” profile of SG – such as nutritional deficiencies, dumping syndrome and increase in bowel frequency [30]. Although this improvement is positive, translating this result into guidance for selecting SG over SR-RYGB (i.e. clinical significance) is limited by two key factors: a 1–2-point HADS score improvement is unlikely to confer a clinically significant improvement (given symptoms are present for scores of 8–21) and HADS is a screening tool – it is not diagnostic [23, 31]. Although the HADS score is objective and validated, monitoring (by a qualified practitioner) of formal psychological diagnoses, severity and treatment (type, dosage) may provide a more clinically relevant evaluation of perioperative mental health. The changes in HADS scores over time highlight that psychological symptoms should be considered perioperatively as recommended in the IFSO guidelines [13].

There are multiple other factors that can impact postoperative psychology which include but are not limited to: recurrent weight gain, body image, body contouring surgery and complications of MBS. Recurrent weight gain can be predicted by and follow psychological symptom scores after MBS [27, 28, 32]. Body contouring surgery is also a contributor to postoperative MBS psychology and intimately linked with body image [33].

At five years, SR-RYGB patients with no pre-existing depressive symptoms had greater weight loss (34.0 kg vs. 23.0 kg, *P* = 0.044). This did not persist to 10 years. Preoperative anxiety symptoms did not impact long-term weight loss either. The link between preoperative psychological symptoms and post-MBS weight loss is poorly understood and published data in this field are inconsistent and lack long-term follow-up beyond five years [34–36].

PF and GH RAND-36 QOL scores significantly improved for both groups over 10 years and were superior with SR-RYGB. Change in RAND-36 scores for each patient over time provides an individualized and objective measure of patient-centred overall health. PF and GH assess patient perceptions of their physical activities and health. Weight loss and resolution of associated medical issues would likely explain the enhanced QOL. Improvement in QOL parameters has been demonstrated in the short-term postoperative period [28, 37–39]. Long term persistence post-MBS is less evident [4, 5, 40].

RE domain scores significantly reduced for the SG group over 10 years and this is in keeping with the published literature [38, 40]. This domain assesses the emotional issues negatively impacting patients (affecting their home, work and daily activities). Recurrent weight gain and post-SG reflux could contribute to this. The pattern of post-MBS weight loss, regain then stability has been linked to corresponding changes within QOL parameters [41, 42]. This highlights the importance of long-term follow-up for identification and treatment (behavioural, medical, surgical) of recurrent weight gain and reflux.

The strengths of this study are that it is a randomised comparison of SG and SR-RYGB with a high follow-up rate (78.4%, excluding the 12 deaths). This paper provides a unique assessment of MBS outcomes by focusing specifically on psychological symptoms and QOL within an exclusive cohort of patients with T2DM and including the ring-augmented variation of RYGB.

There are some limitations to consider. There was no control group thus limiting the comparative strength of the two groups. The study was underpowered for patient-centred outcomes as it was not designed to detect a difference in these at 10-years postoperative. This may particularly be relevant to the improved anxiety scores within the SG group whereby the relationship between a statistically significant result and clinical significance is not completely understood. The use of the HADS screening tool allowed comparison for the same patient over time but clinical relevance is not easily transferable – limiting the ability of these data to guide recommendations for specific MBS. Finally, information regarding bariatric-specific postoperative QOL (e.g. dysphagia, vomiting) would allow a more thorough patient-centred comparison of SG and SR-RYGB. Using a specific validated score (such as the bariatric analysis and reporting outcome system) would allow a more holistic assessment [43, 44]. With suitably powered future studies focusing on psychological symptoms and QOL, the approach to MBS selection for global obesity management could be tailored to individual patients which may improve outcomes from a patient and disease perspective.

## Conclusion

In the context of this study, SR-RYGB is superior to SG with regard to weight loss, diabetes remission and the physical components of QOL assessment. Pre-existing depression and anxiety symptoms do not clearly and meaningfully impact long-term weight loss. However, SG may result in reduced long-term anxiety symptoms (as well as improved QOL). Future research should be powered towards detecting differences in these patient-centred outcomes to offer an individualised approach to obesity management.

## Data Availability

Datasets generated and analysed during the study can be requested directly from the publishing authors on reasonable request and would need to be approved at a local hospital level.
